# King Edward’s Illness

**Published:** 1902-08

**Authors:** 


### KING EDWARD'S ILLNESS.

THE first gentleman in England, so long the glass of fashion and the mould of form, has been true to his tendencies, even to the point of being smitten with the fashionable ailment of appendicitis. The case as narrated in the secular press had nothing in the way of unusual features connected with it. It appears to have been an appendicitis, with abscess formation and destruction of the appendix. The operation was simply that of evacuation of the pus and drainage of the abscess cavity. England is to be congratulated upon the fortunate termination of an episode in its history which came very near being a tragedy, and which brought such overwhelming disappointment, dismay and pecuniary loss upon the people by its untimely interruption of the coronation.

The greatest danger that was apprehended was in the general condition of the royal patient—not so much the disease, for that is of low mortality. Old, fat, bull-necked and plethoric he was an ideally poor subject for an anesthetic, but underlying all this there seems to have been a considerable reserve of strength of constitution which doubtless contributed largely to the final successful issue of the case.

The ailments and diseases which have from time to time been attributed to the present king would make a very formidable list. Cancer of the larynx, based on a laryngeal catarrh and an habitually harsh and husky voice, chronic dyspepsia and constipation, Bright's disease and whispers of less reputable complaints have all had their turn with rumor. But the continued vigor of the king seems to have set at naught the imaginings of idle *quid nuncs.* It is the ardent hope of the American people that the successor of the venerable Victoria will live long and add wisely and well to the luster and fame of Great Britain.

---

THE following from the *Philadelphia Medical Journal* shows one way of limiting the spread of contagious disease :

Charles V. Chapin makes the following suggestions for the teaching of cleanliness among school children in his article on "One Way to Fight Contagion:"

"1. Not to spit, it is rarely necessary—to spit on a slate, floor, or sidewalk is an abomination.

"2. Not to put the fingers into the mouth.

"3. Not to pick the nose.

"4. Not to wet the finger with saliva in turning the leaves of a book.

"5. Not to put pencils in the mouth or moisten them with the lips.

"6. Not to put money into the mouth.

"7. Not to put pins into the mouth.

"8. Not to put anything in the mouth except food and drink.

"9. Not to swap apple cores, candy, chewing-gum half-eaten, whistles, or bean blowers, or anything that is habitually put into the mouth.

"10. Teach the children to wash the hands and face often ; see that they keep them clean. If a child is coming down with a communicable disease it is reasonable to believe that there is less chance of infecting persons and things if the hands and face are washed clean and are not daubed with secretions of the nose and mouth.

"11. Teach the children to turn the face aside when coughing and sneezing, if they are facing another person.

"12. Children should be taught that their bodies are their own private possessions, that personal cleanliness is a duty, that the mouth is for eating and speaking and should not be used as a pocket, and the lips should not take the place of fingers."

